# Association Between Allostatic Load and Delirium in ICU Patients: A Retrospective Analysis of the MIMIC-IV Database

**DOI:** 10.3390/jcm14113916

**Published:** 2025-06-03

**Authors:** Yubei Zhou, Yuenan Ni, Lan Lan, Huajing Wan, Fengming Luo

**Affiliations:** 1 Department of Respiratory and Critical Care Medicine, West China School of Medicine and West China Hospital, Sichuan University, Chengdu 610041, China; zybhh1004@163.com (Y.Z.); vivian940305@foxmail.com (Y.N.); lanlanay@163.com (L.L.); wanhuajing1974@wchscu.cn (H.W.); 2 Laboratory of Pulmonary Immunology and Inflammation, Frontiers Science Center for Disease-Related Molecular Network, West China Hospital, Sichuan University, Chengdu 610041, China; 3 Department of Respiratory Care, West China School of Medicine and West China Hospital, Sichuan University, Chengdu 610041, China

**Keywords:** allostatic load, delirium, intensive care medicine

## Abstract

**Background**: Allostatic load reflects the cumulative physiological effects of chronic and repeated stress on the body and is associated with dysregulation of multiple systems. This study aimed to examine the association between the allostatic load score (ALS) and the development of delirium in intensive care unit (ICU) patients. **Method**: The adult patients from the Medical Information Mart for Intensive Care (MIMIC-IV) database were screened and included in this study. Allostatic load was scored by hemoglobin A1c, high-density lipoprotein, total cholesterol, systolic blood pressure, diastolic blood pressure, body mass index, C-reactive protein, and serum albumin, and varied from 0 to 8. Restricted cubic spline and multivariate logistic regression were used to assess the relationship between ALS and delirium risk in the ICU. The threshold of the ALS was determined by the decision tree approach. A sensitivity analysis was also conducted. **Results**: A total of 656 patients were included in the study, and the incidence of delirium was 50.6% (n = 332). In a fully adjusted restricted cubic spline model, an increase in ALS was linearly positively correlated with the occurrence of delirium in the ICU (*p*-overall = 0.039, *p*-nonlinear = 0.506). The threshold for ALS was determined to be 3. ALS ≥ 3 was associated with increased delirium rates (*p* < 0.001), longer hospital stays (*p* < 0.001), and higher in-hospital mortality (*p* = 0.002). Subgroup analyses revealed no significant interactions (all *p* values for interactions > 0.05). **Conclusions**: Higher ALS was linearly associated with increased risk of ICU delirium. An ALS ≥ 3 identified patients with greater delirium incidence, longer hospital stays, and higher mortality.

## 1. Introduction

Delirium is an acute form of brain dysfunction characterized by sudden confusion, a fluctuating course, inattention, and altered consciousness [1]. The incidence of delirium is notably high in the intensive care unit (ICU) about approximately 20% to 50% [2]. Among patients of any age admitted to the ICU and requiring mechanical ventilatory support, the prevalence of delirium is as high as 80% [3]. In the ICU, delirium independently predicts several critical outcomes, including higher healthcare costs, extended ICU and hospital stays, increased mortality, and long-term cognitive impairment. However, delirium is a multifactorial disease with q complex pathological mechanism. There are various interactions between its aetiologia and precipitating factors, making it difficult to explain its etiology or phenomenological manifestations with a single theory [4].

In the short term, the body’s stress response is adaptive. However, prolonged or excessive activation of these compensatory mechanisms may lead to maladaptation, physiological dysregulation, or suboptimal forms of adaptation [5]. Allostatic load (AL) refers to the cumulative physiological burden imposed on the body due to prolonged exposure to chronic or repeated stress [6,7,8]. When chronic stress exceeds coping capacity, AL increases as the stress response system is repeatedly overactivated, causing cardiovascular, metabolic, and immune dysfunction that may lead to serious health conditions. Previous studies have shown that a higher AL is associated with various malignant tumors, cardiovascular diseases, endocrine disorders, depression, and other diseases [9,10,11,12,13]. Current research typically quantifies AL by calculating the allostatic load score (ALS). As a comprehensive parameter used to measure the impact of stress on the physiological system, the calculation of the ALS usually includes three types of key indicators: cardiovascular, metabolic, and immune [14,15]. Studies have shown that the pathogenesis of delirium may be associated with stress-related mechanisms [16,17]. The integrated stress response contributing to delirium includes the activation of the sympathetic nervous system, metabolic factors, and the immune system [18]. The overlap of key systems and factors associated with AL and delirium suggests that the pathophysiological changes leading to delirium may share common features with changes in AL. However, the exact correlation between ALS and the occurrence of ICU delirium has not been confirmed, which requires further research.

The purpose of this study was to investigate the relationship between AL and delirium in ICU patients. It is expected to enhance the early detection of delirium in the ICU, provide new mechanistic insights into delirium pathophysiology, and support the development of delirium prevention methods based on AL in intensive care settings.

## 2. Materials and Methods

### 2.1. Data Sources

This study is a retrospective analysis using the Medical Information Mart for Intensive Care (MIMIC-IV version 3.0) database. The MIMIC-IV is a collaboration between Beth Israel Deaconess Medical Center (BIDMC) and the Massachusetts Institute of Technology (MIT) that contains data on ICU patients admitted to BIDMC from 2008–2022 [19,20]. One author (Yuenan Ni) complied with the requirements for access to the MIMIC-IV database and was responsible for the data extraction.

### 2.2. Cohort Selection

ALS includes eight indicators: hemoglobin A1c (HbA1c), high-density lipoprotein (HDL), total cholesterol, systolic blood pressure (SBP), diastolic blood pressure (DBP), body mass index (BMI), C-reactive protein (CRP), and serum albumin (ALB). The inclusion criteria included (1) age > 18 years, (2) no language barrier, and (3) patients with indicators for calculating ALS. The exclusion criteria included (1) an ICU length of stay of less than 24 h; (2) major data missing or incorrect; and (3) a history of schizophrenia or dementia. We ultimately enrolled 656 patients and divided them into two groups on the basis of whether they developed delirium in the ICU.

### 2.3. Data Collection

In this study, we used PostgreSQL14 and pgAdmin 4 (version 8.1) to extract the data. The extracted demographic characteristics included the patient’s age, sex, race, BMI, and Sequential Organ Failure Assessment (SOFA) score. The vital signs included body temperature, heart rate, respiratory rate, SBP, DBP, MBP, and peripheral capillary oxygen saturation (SpO_2_). Laboratory test results, including HBA1c, albumin, C-reactive protein (CRP), total cholesterol, high-density lipoprotein (HDL), white blood cell (WBC), hemoglobin, platelet, creatinine, blood urea nitrogen, anion gap (AG), and international standardized ratio (INR) levels, were collected within 24 h of admission to the ICU. The comorbidities included myocardial infarct, congestive heart failure, peripheral vascular disease, cerebrovascular disease, chronic pulmonary disease, rheumatic disease, peptic ulcer disease, liver disease, diabetes, renal disease, malignant tumors, and sepsis. In addition, major events during the patient’s ICU stay, including mechanical ventilation (MV), reintubation within 48 h, length of ICU stay, length of hospital stay, and in-hospital death, were recorded.

In this study, the algorithm of ALS employs the most widely used computational method currently [15]. The algorithm for ALS converts each biomarker into a dichotomous variable on the basis of the high-risk quartile, and the scores are added together as ALS. If the biomarker is in the high-risk range (i.e., the highest quartile), it receives 1 point, and if it is not in the high-risk range (i.e., the lowest 3 quartiles), it receives 0 points. Among them, the serum ALB and HDL levels are in the lowest quartile of the high-risk range, with a score of one point. The ALS score is between 0 and 8.

### 2.4. Outcomes

The main outcome of this study was the occurrence of delirium in the ICU. Two methods are used to assess delirium in patients. The first method uses the ICU Confusion Assessment Method (CAM-ICU), an accurate assessment tool for screening for delirium in critically ill patients [21]. Second, the International Classification of Diseases, Ninth Revision, Clinical Modification (ICD-9-CM) and Tenth Revision (ICD-10-CM) were used to identify patients with a formal diagnosis of delirium. Specifically, ICD-9-CM code 293.0 (Acute delirium due to conditions classified elsewhere) and ICD-10-CM code F05 (Delirium due to known physiological condition) were used [22,23]. Patients identified as delirious by either of these assessment methods are categorized into the delirium group, whereas those who do not meet the criteria are classified into the non-delirium group. The secondary outcomes of this study included (1) reintubation within 48 h after extubation; (2) length of stay in the ICU (ICU LOS, in days); (3) total hospital length of stay (hospital LOS, in days); and (4) in-hospital death.

### 2.5. Statistical Analysis

Patients were divided into two groups on the basis of whether delirium developed in the ICU. Categorical variables were compared via chi-square tests. For continuous variables, normality was assessed via the Shapiro-Wilk test. Normally distributed variables were analyzed via Student’s *t* test and are presented as the means ± standard deviations, whereas non-normally distributed variables were analyzed via the Wilcoxon rank sum test and are presented as medians and quartiles with interquartile ranges. Statistical significance was set at *p* < 0.05 (two-sided).

Variables showing significant differences initially were further evaluated via least absolute shrinkage and selection operator (LASSO) regression for feature selection and model simplification. Variables with a variance inflation factor (VIF) ≥ 5 were excluded to avoid multicollinearity. We used univariate and multivariate restricted cubic spline (RCS) regressions to assess possible nonlinear relationships between ALS and the risk of delirium in ICU patients. Model A was unadjusted; Model B was adjusted for sex, age, race, hemoglobin, platelet, WBC, creatinine, blood urea nitrogen, AG, and INR; Model C was adjusted for Model B variables plus comorbidities: myocardial infarct, congestive heart failure, peripheral vascular disease, cerebrovascular disease, chronic pulmonary disease, rheumatic disease, peptic ulcer disease, liver disease, renal disease, malignant tumor, sepsis, and mechanical ventilation. A stepwise binary logistic regression was then performed using selected variables and clinically relevant factors to examine the independent associations between ALS and delirium. The optimal ALS cutoff value was determined via decision tree using the “rpart” package (version 4.1.24) in R. The patients were then divided into a high ALS group and a low ALS group due to the threshold, and the differences in outcomes between the two groups were tested.

For robustness verification, we conducted sensitivity analyses excluding patients with sepsis or who were on mechanical ventilation. Stratified analyses, including age, sex, race, sepsis, and the requirement for invasive mechanical ventilation, were used to assess the effects of ALS on different subgroups. To minimize bias, among the variables not included in ALS, only those with less than 10% missing data were retained. Missing data were imputed via the multiple imputation method using the “mice” package (version 3.17.0) in R. All analyses were conducted via R (version 4.3.2) and SPSS (version 24.0; IBM SPSS Statistics, Armonk, NY, USA).

## 3. Results

### 3.1. Study Population

**Figure 1:**
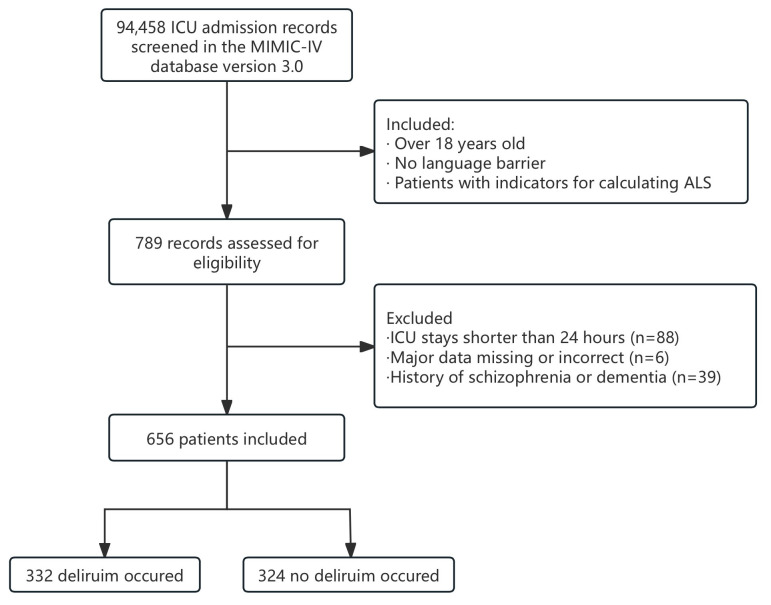
Flowchart of patient selection from the MIMIC-IV database. Abbreviations: ICU, intensive care unit; MIMIC-IV, Medical Information Mart for Intensive Care IV; ALS, allostatic load score.

As shown in Figure 1, a total of 94,458 ICU admission records were screened from the MIMIC-IV database. A total of 656 patients were ultimately included in the study, with 332 patients (50.6%) developing delirium and 324 patients (49.4%) without delirium. Table 1 summarizes the demographic and clinical characteristics of the patients. The median age of the enrolled patients was 66 years (IQR, 54–76 years), with no significant difference between the two groups (*p* = 0.696). The study population consisted of 291 females (44.4%) and 365 males (55.6%), 374 (57%) of whom were white, with significant differences in distribution between the delirium and non-delirium groups (*p* < 0.001). The median ALS was 2 (IQR, 1–3). Mechanical ventilation was required for 283 patients (43.1%), with 23 patients (3.5%) being reintubated within 48 h after extubation. The median ICU LOS was 5 days (IQR, 2–10), while the median hospital LOS was 16 days (IQR, 8–28). The in-hospital mortality rate was 12.5% (n = 82).

### 3.2. ALS and Other Risk Factors

Compared with patients in the non-delirium group, patients in the delirium group had a significantly greater proportion of ALS ≥ 3 (132 (39.8%) vs. 87 (26.9%), *p* < 0.001), an increased prevalence of sepsis (247 (74.4%) vs. 112 (34.6%), *p* < 0.001), a decreased prevalence of rheumatic disease (6 (1.8%) vs. 19 (5.9%), *p* = 0.012), and a greater requirement for mechanical ventilation (203 (61.1%) vs. 80 (24.7%), *p* < 0.001). There were statistically significant differences in race, SOFA score, heart rate, respiratory rate, body temperature, SpO_2_, ALB, WBC count, hemoglobin, blood urea nitrogen, anion gap, and INR between the delirium group and the non-delirium group (*p* < 0.05).

**Figure 2:**
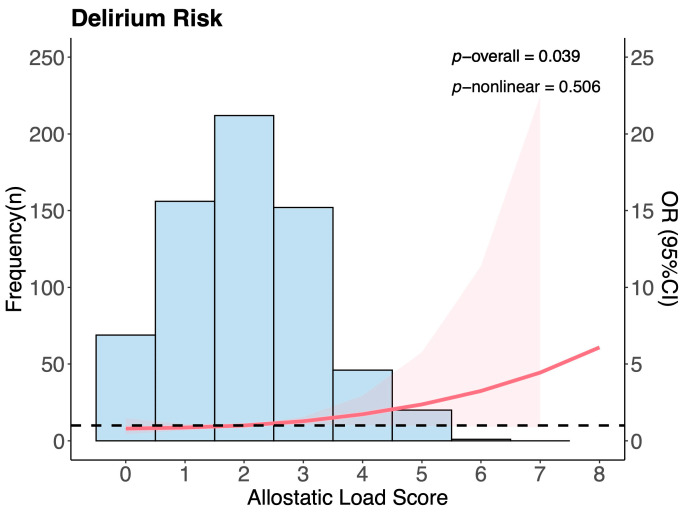
Fully adjusted restricted cubic spline regression analysis (model C) between ALS level and delirium risk in ICU patients. The model C was adjusted for sex, age, race, laboratory test parameters (hemoglobin, platelet, WBC, creatinine, blood urea nitrogen, AG, INR), comorbidities (myocardial infarct, congestive heart failure, peripheral vascular disease, cerebrovascular disease, chronic pulmonary disease, rheumatic disease, peptic ulcer disease, liver disease, renal disease, malignant tumor, sepsis), and mechanical ventilation. Blue bars represent the frequency distribution of ALS within the study population. The red solid line indicates the fitted spline curve, with the pink shaded area representing the 95% CI. The horizontal dashed line represents the reference level (OR = 1.0). Abbreviations: ALS, allostatic load score; ICU, intensive care unit; WBC, white blood cell count; AG, anion gap; INR, international normalized ratio; CI, confidence interval.

To assess potential overlap between ALS components and the SOFA score, we performed a VIF analysis. All VIFs were below 5, indicating no significant multicollinearity and suggesting that ALS and SOFA score are statistically independent (Supplementary Table S1). After LASSO regression (Supplementary Table S2) and VIF-based variable selection (Supplementary Table S3) were performed, the following factors were included in the subsequent multivariable analysis: ALS, race, temperature, SOFA score, respiratory rate, SpO_2_, WBC, hemoglobin, AG, sepsis, rheumatic disease, and mechanical ventilation. The linear relationship between ALS and the incidence of delirium in the ICU was analyzed via RCS. The unadjusted RCS model A (*p*-overall = 0.002, *p*-nonlinear = 0.512), the RCS model B adjusted for sex, age, race, and laboratory test parameters (*p*-overall = 0.008, *p*-nonlinear = 0.956), and the fully adjusted RCS model C (*p*-overall = 0.039, *p*-nonlinear = 0.506) all demonstrated a linear relationship between ALS and the risk of ICU delirium (Figure 2).

The results of the binary logistic regression analysis are presented in Table 2. Higher ALS was an independent risk factor for ICU delirium after adjustment (OR = 1.20, 95% CI: 1.03–1.40, *p* = 0.016). Similarly, elevated body temperature (OR: 1.05, 95% CI: 1.01–1.09, *p* = 0.021), a greater AG (OR: 1.06, 95% CI: 1.01–1.11, *p* = 0.029), sepsis (OR: 3.22, 95% CI: 2.21–4.69, *p* < 0.001), and the need for mechanical ventilation (OR: 3.48, 95% CI: 2.38–5.08, *p* < 0.001) were also independent risk factors for delirium. Patients with rheumatic diseases (OR: 0.32, 95% CI: 0.11–0.91, *p* = 0.032) or white individuals (OR: 0.61, 95% CI: 0.42–0.87, *p* = 0.007) had a lower risk of developing delirium.

To compare the effects of high ALS and low ALS on the occurrence of delirium in the ICU, based on a decision tree approach, the optimal cut-off point was automatically determined by recursively splitting the data to maximize sample purity. The analysis identified a score of 3 as the optimal threshold for the ALS, which was then used for subsequent analyses (Supplementary Figure S1). Due to the threshold of ALS, patients were stratified into two groups: the high-ALS group (ALS ≥ 3, n = 219) and the low-ALS group (ALS ≤ 2, n = 437). Compared with the low-ALS group, the high-ALS group had significantly greater rates of delirium (132 (60.3%) vs. 200 (45.8%), *p* < 0.001), longer hospital LOS (18 (10, 33) vs. 15 (7, 27) days, *p* = 0.001), and increased in-hospital mortality (40 (18.3%) vs. 42 (9.6%), *p* = 0.002). The rates of reintubation within 48 h after extubation, length of ICU stay, and use of mechanical ventilation were not significantly different between the two groups (all *p* > 0.05) (Table 3).

### 3.3. Sensitivity Analysis

**Figure 3:**
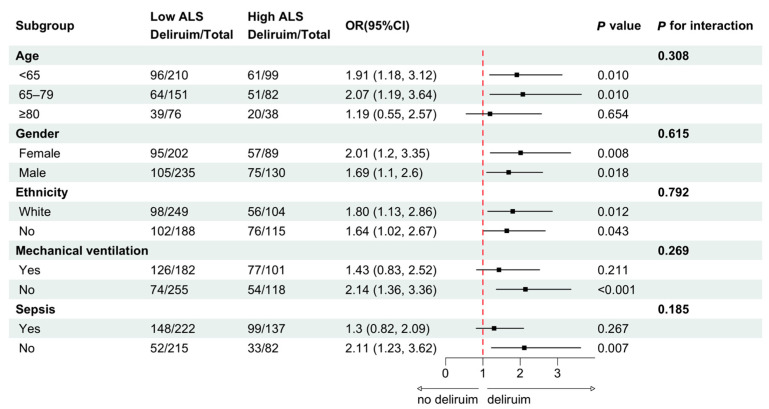
Subgroup interaction analysis to assess the association between ALS and delirium occurrence in ICU patients. Black squares represent the point of the OR, with horizontal lines indicating 95% CI. Abbreviations: ALS, allostatic load score; OR, odds ratio; CI, confidence interval; ICU, intensive care unit.

To verify the robustness of the main results, a sensitivity analysis was performed. After patients with sepsis (Supplementary Tables S4 and S5) or those requiring mechanical ventilation (Supplementary Tables S6 and S7) were excluded, the significant association between ALS and delirium persisted. Furthermore, high ALS was still associated with a longer hospital LOS. On the basis of subgroup analysis, patients were stratified by age, sex, race, need for mechanical ventilation, and sepsis status. The association between ALS and the risk of developing ICU delirium did not significantly differ across all subgroups (all *p* values for interactions > 0.05) (Figure 3).

## 4. Discussion

This study confirmed a clear association between ALS and the risk of delirium in ICU patients. The main findings of this study can be summarized as follows: Patients with delirium in the ICU had a significantly higher rate of ALS ≥ 3 than those without delirium. The increase in the ALS was linearly correlated with the risk of delirium in the ICU and remained an independent risk factor for delirium after adjusting for potential confounders. Patients with an ALS ≥ 3 had a greater incidence of delirium, longer hospital stays, and increased in-hospital mortality.

AL refers to the wear and tear of the body caused by long-term and repeated exposure to pressure [7]. When the body is threatened, the brain initiates the sympathetic adrenal medulla (SAM) axis and hypothalamic-pituitary-adrenal (HPA) axis [24], leading to the release of stress hormones. These biomarkers are referred to as primary mediators [25]. The synergy of these molecules can have a primary effect on cell function, disrupting the body’s homeostatic regulatory mechanisms. Over time, the body activates compensatory mechanisms in response to fluctuations in the primary mediator, which can lead to changes in secondary mediators. The final stage of AL progression is allostatic overload, which exceeds the body’s ability to regulate and ultimately leads to disorders and diseases [15,26].

During the path from psychosocial stress to disease, the primary mediators mainly include epinephrine, norepinephrine, and cortisol, while the secondary mediators mainly refer to the subclinical changes that occur under physiological dysfunction. Due to the relatively difficult clinical measurement of the primary mediators and their susceptibility to interference from exogenous hormones, in order to enhance clinical feasibility the biomarkers selected for this study were secondary mediators, which are the ones that were most used in previous research on ALS assessment [14] and align with those reported in the existing literature [27]. As an immune marker, CRP is a well-established indicator of inflammation that reflects the systemic inflammatory status of critically ill patients. For metabolic markers, HbA1c, ALB, and BMI were chosen, as they provide insights into glycemic control, nutritional status, and overall metabolic health, which are the key factors of chronic stress and metabolic dysfunction. Cardiovascular markers included SBP, DBP, HDL, and total cholesterol. Blood pressure directly measures cardiovascular responses, while HDL and total cholesterol are essential indicators of lipid metabolism, closely associated with cardiovascular risk.

Delirium is the most common manifestation of brain dysfunction associated with serious critical illness [28]. The underlying pathophysiological mechanisms of delirium remain complex and incompletely understood, and the etiology is unclear. There are no effective drugs to prevent or treat delirium [29], and there is currently no evidence to support any single biomarker as a diagnostic or prognostic marker [30,31].

The risk of delirium is determined by predisposing risk factors and precipitating risk factors. Predisposing risk depends on long-term stimuli, that is, the background characteristics of the patient, such as increasing age, comorbidities, and mental illness [32]. Conversely, precipitating risk is usually determined by acute, short-term stimuli, such as sepsis and surgery [29]. Therefore, the occurrence of delirium involves complex interactions between the brain and multiple systems. Previous studies have suggested that the mechanisms of delirium may include brain energy metabolism, inflammation, stress and neurotransmitter imbalance, neuroanatomical substrates, and failure of network connectivity [29]. The cerebral metabolic insufficiency hypothesis [33] proposes that a lack of oxygen or glucose impairs brain function. When insulin resistance occurs, elevated blood glucose with decreased brain glucose utilization can lead to metabolic insufficiency, potentially increasing the risk of delirium [29]. When systemic inflammation acts as a stressor, inflammatory signals can cross the blood-brain barrier, triggering underlying delirium exacerbation [34]. Studies have demonstrated a synergistic effect between inflammation and stress. Chronic unpredictable stress can exacerbate the inflammatory response in the brain [35]. As a comprehensive indicator of the state of the cardiovascular, metabolic, and immune systems, AL may overlap with the pathophysiology of delirium. Compared with a single biomarker, AL can better explain how chronic and long-term stress accumulates and affects multiple biological systems, providing a more detailed perspective on the potential mechanisms of delirium. Currently, the prevention and screening of ICU delirium rely on validated tools, with the most widely used being the CAM-ICU and the Intensive Care Delirium Screening Checklist (ICDSC). These tools mainly focus on assessing patients’ consciousness and mental status to identify delirium [36]. However, they are largely based on clinical observation and may be influenced by the evaluator’s experience and the patient’s cooperation. For instance, accurately assessing patients who are under sedation or analgesia might be more challenging. ALS monitoring offers an objective approach by integrating multiple biomarkers, providing an additional physiological dimension to the existing methods. These objective data enhance early delirium detection and risk assessment, complementing traditional methods that rely on subjective clinical evaluation.

Rigney [37] reported that the primary mediator scores of AL, including urinary cortisol, norepinephrine, and epinephrine, and serum dehydroepiandrosterone sulfate, can predict the incidence of delirium in patients aged 65 years and older (OR: 2.54, 95% CI: 1.12–5.79; *p* < 0.05). However, monitoring these indicators requires many more medical resources and has higher laboratory testing costs, and many ICU patients receive exogenous hormones, norepinephrine, and epinephrine, making the accurate determination of the endogenous level of patients challenging. Compared with previous studies, this study used a large database with a larger sample size and calculated the ALS using relevant indicators of secondary mediators, which are more readily available in clinical practice. Sensitivity and subgroup analyses were performed to ensure the robustness of the results. In addition, we classified ALS patients and reported that patients with higher ALS scores had poorer short-term outcomes.

This study has several limitations. First, it is a retrospective study based on a database, which may introduce biases and difficulties in inferring causal relationships. Although our findings reveal a significant association between ALS and ICU delirium, the design of the study prevents us from establishing causality. Therefore, these results should be interpreted as correlational rather than causal. Additionally, this study only uses the MIMIC-IV database, the sample size is relatively small, and the results were not verified externally. This may limit the external validity of the results, making it impossible to confirm their applicability to other populations. Second, due to the lack of relevant records, we were unable to evaluate the impact of sedative medications and sleep disruption on the development of delirium, although both are considered important contributing factors. In addition, we could not assess the duration of delirium, which is a critical factor in evaluating patient prognosis. Third, there is also a potential risk of misclassification in the identification of delirium, as CAM-ICU assessment relies on the judgment of clinical staff, which may be subjective or miss hypoactive cases, while ICD coding depends on accurate documentation. Fourth, multiple imputation was applied to a small subset of laboratory test results with less than 10% missingness. However, all variables required for calculating the ALS and all outcome measures were complete, so the impact of imputation on the overall study findings is expected to be minimal. Fifth, in this study, ALS was established based on the data within 24 h after the patients were admitted to the ICU. For some acute diseases that could affect the components of ALS, the ALS we calculated might not accurately reflect the chronic stress of these patients. Sixth, there is currently no recognized ALS algorithm and the markers selected in the existing studies are slightly different. In the future, more standardized and universally recognized biomarkers and calculation methods will be needed.

To address these limitations, future studies should conduct multicenter, prospective research in patients from different regions and different types of hospitals to evaluate the accuracy and stability of ALS in predicting ICU delirium. Moreover, validation in different populations will help promote the application of this scoring system in a wider range of clinical settings.

## 5. Conclusions

In conclusion, the increase in ALS was linearly correlated with the risk of delirium in the ICU and associated with clinical outcomes of critically ill patients. The optimal cutoff value of ALS was found to be 3. These findings suggest that ALS enables early identification of delirium in the ICU and will be a promising indicator for the management of delirium and improvement in outcomes in critically ill patients. Further large-scale prospective studies are warranted to validate these findings.

## Figures and Tables

**Table 1:** Baseline characteristics of patients grouped by non-delirium or delirium status in the ICU.

Variables	Overall	Non-Delirium	Delirium	*p* Value
Total	n = 656	n = 324	n = 332	
Age (year)	66 (54, 76)	66 (53, 76)	66 (55, 75)	0.696
Female Sex (n, %)	291 (44.4)	139 (42.9)	152 (45.8)	0.458
Race (n, %)				<0.001 *
White	374 (57)	209 (64.5)	165 (49.7)	
Others	282 (43)	115 (35.5)	167 (50.3)	
BMI (kg/m^2^)	27.29 (23.48, 31.73)	27.3 (23.51, 31.76)	27.1 (23.29, 31.73)	0.615
ALS (score)	2 (1, 3)	2 (1, 3)	2 (1, 3)	<0.001 *
ALS ≥ 3 (n, %)	219 (33.4)	87 (26.9)	132 (39.8)	<0.001 *
SOFA (score)	4(2, 6)	3(1, 5)	4(3, 7)	<0.001 *
Heart rate (beats/min)	83 (73, 93)	80 (72, 92)	86 (73, 96)	0.012 *
Respiratory rate (times/min)	19 (17, 22)	19 (17, 21)	20 (18, 22)	<0.001 *
Temperature (°C)	36.9 (36.7, 37.2)	36.9 (36.7, 37.1)	37 (36.7, 37.3)	<0.001 *
SBP (mmHg)	129.7 (101.65, 155.76)	128.59 (100.84, 156.34)	130.78 (102.44, 159.12)	0.316
DBP (mmHg)	74 (63, 85)	73 (63, 83.75)	74 (63, 87)	0.331
MBP (mmHg)	83 (75, 92)	83 (75.25, 92.75)	82 (75, 92)	0.580
SpO_2_ (%)	97 (96, 98)	97 (96, 98)	98 (96, 99)	0.001 *
**Laboratory tests**				
HbA1c (%)	5.7 (5.3, 6.5)	5.6 (5.3, 6.4)	5.8 (5.3, 6.58)	0.528
Serum albumin (g/dL)	3.4 (2.9, 3.8)	3.5 (3.1, 3.9)	3.3 (2.73, 3.7)	<0.001 *
C-reactive protein (mg/dL)	34.65 (6.5, 99.5)	35.7 (6.75, 91.4)	33.25 (6.25, 107.25)	0.870
Cholesterol (mg/dL)	143.5 (113, 180.75)	143 (113, 173.75)	146 (113, 188.75)	0.248
HDL(mg/dL)	41 (29, 55)	40.5 (28, 53)	42 (30, 57)	0.105
WBC (10^9^/L)	10.65 (7.96, 14.49)	9.85 (7.52, 13.63)	11.43 (8.81, 15.25)	<0.001 *
Hemoglobin(g/dL)	11.1 (9.15, 12.95)	11.4 (9.45, 13.1)	10.7 (8.81, 12.65)	0.034 *
Platelet (10^9^/L)	207 (156, 273)	206.5 (162.5, 269)	207.5 (151, 278.38)	0.569
Creatinine (mg/dL)	1 (0.75, 1.5)	0.95 (0.7, 1.4)	1.05 (0.75, 1.75)	0.014 *
Blood urea nitrogen (mg/dL)	18 (12.5, 30)	17 (12, 27)	20 (13, 35)	0.003 *
AG (mEq/L)	14 (12, 16.5)	14 (12, 16)	14.5 (12, 17)	0.044 *
INR	1.2 (1.1, 1.45)	1.2 (1.1, 1.4)	1.2 (1.1, 1.5)	0.043 *
**Comorbidities**				
Myocardial infarct (n, %)	144 (22)	69 (21.3)	75 (22.5)	0.689
Congestive heart failure (n, %)	234 (35.7)	109 (33.6)	125 (37.7)	0.284
Peripheral vascular disease (n, %)	80 (12.2)	44 (13.6)	36 (10.8)	0.341
Cerebrovascular disease (n, %)	423 (64.5)	206 (63.6)	217 (65.4)	0.693
Chronic pulmonary disease (n, %)	115 (17.5)	54 (16.7)	61 (18.4)	0.565
Rheumatic disease (n, %)	25 (3.8)	19 (5.9)	6 (1.8)	0.012 *
Peptic ulcer disease (n, %)	19 (2.9)	8 (2.5)	11 (3.3)	0.681
Liver disease (n, %)	78 (11.9)	33 (10.2)	45 (13.6)	0.183
Diabetes (n, %)	232 (35.4)	104 (32.1)	128 (38.6)	0.084
Renal disease (n, %)	148 (22.6)	66 (20.4)	82 (24.7)	0.185
Malignant tumor (n, %)	58 (8.8)	34 (10.5)	24 (7.2)	0.141
Sepsis (n, %)	359 (54.7)	112 (34.6)	247 (74.4)	<0.001 *
**Events**				
Mechanical ventilation (n, %)	283 (43.1)	80 (24.7)	203 (61.1)	<0.001 *
Reintubation within 48 h (n, %)	23 (3.5)	7 (2.2)	16 (4.8)	0.064
ICU LOS (day)	5 (2, 10)	3 (2, 5)	8 (4, 15)	<0.001 *
Hospital LOS (day)	16 (8, 28)	11 (5, 19)	22 (12, 37)	<0.001 *
In-hospital death (n, %)	82 (12.5)	29 (9.0)	53 (16.0)	0.009 *

Abbreviations: BMI, body mass index; ALS, allostatic load score; SOFA, Sequential Organ Failure Assessment; SBP, systolic blood pressure; DBP, diastolic blood pressure; MBP, mean blood pressure; SpO_2_, peripheral capillary oxygen saturation; HbA1c, hemoglobin A1c; HDL, high-density lipoprotein; WBC, white blood cell count; AG, anion gap; INR, international normalized ratio; ICU, intensive care unit; LOS, length of stay. * *p* < 0.05.

**Table 2:** Multivariate logistic regression analysis of delirium occurrence in the ICU.

Variable	OR (95% CI)	*p* Value
Allostatic load score	1.20 (1.03, 1.40)	0.016 *
Temperature (°C)	1.05 (1.01, 1.09)	0.021 *
Respiratory rate (times/min)	1.04 (0.99, 1.09)	0.087
Race (n, %)	0.61 (0.42, 0.87)	0.007 *
AG (mEq/L)	1.06 (1.01, 1.11)	0.029 *
Sepsis (n, %)	3.22 (2.21, 4.69)	<0.001 *
Rheumatic disease (n, %)	0.32 (0.11, 0.91)	0.032 *
Mechanical ventilation (n, %)	3.48 (2.38, 5.08)	<0.001 *

Abbreviations: ICU, intensive care unit; OR, odds ratio; CI, confidence interval; AG, anion gap. * *p* < 0.05.

**Table 3:** Outcomes of the low-ALS group and the high-ALS group.

Variable	Overall	ALS ≤ 2	ALS ≥ 3	*p* Value
	n = 656	n = 437	n = 219	
Delirium (n, %)	332 (50.6)	200 (45.8)	132 (60.3)	<0.001 *
Reintubation within 48 h (n, %)	23 (3.51)	15 (3.43)	8 (3.65)	0.885
ICU LOS (day)	5 (2, 10)	5 (2, 10)	5 (2, 11)	0.546
Hospital LOS (day)	16 (8, 28)	15 (7, 27)	18 (10, 33)	0.001 *
In-hospital death (n, %)	82 (12.5)	42 (9.6)	40 (18.3)	0.002 *
Mechanical ventilation (n, %)	283 (43.1)	182 (41.6)	101 (46.1)	0.276

Abbreviations: ALS, allostatic load score; ICU, intensive care unit; LOS, length of stay. * *p* < 0.05.

## Data Availability

This study conducted an analysis of publicly available datasets. These data can be accessed here: https://mimic.mit.edu/ (accessed on 21 April 2025).

## Supplementary Materials

The following supporting information can be downloaded at: https://www.mdpi.com/article/10.3390/jcm14113916/s1, Table S1: The variance inflation factors for ALS components and the SOFA score; Table S2: Result of lasso regression; Table S3: The variance inflation factor for variables that have a significant effect on delirium after lasso regression screening; Table S4: The association between ALS and risk of delirium in the ICU after excluding patients with sepsis (n = 297); Table S5: Outcome of patients with low and high ALS after excluding patients with sepsis (n = 297); Table S6: The association between ALS and risk of delirium in the ICU after excluding patients with mechanical ventilation (n = 373); Table S7: Outcome of patients with low and high ALS after excluding patients with mechanical ventilation (n = 373); Figure S1: Decision Tree for Identifying Optimal ALS Threshold for Delirium.

## Abbreviations

AL, allostatic load; ICU, intensive care unit; MIMIC-IV, Medical Information Mart for Intensive Care; RCS, restricted cubic spline; ALS, allostatic load score; BIDMC, Beth Israel Deaconess Medical Center; HBA1c, hemoglobin A1c; HDL, high-density lipoprotein; SBP, systolic blood pressure; DBP, diastolic blood pressure; BMI, body mass index; CRP, C-reactive protein; SOFA, the Sequential Organ Failure Assessment; SpO_2_, peripheral capillary oxygen saturation; WBC, white blood cell count; AG, anion gap; INR, international standardized ratio; MV, mechanical ventilation; LASSO, least absolute shrinkage and selection operator; VIF, variance inflation factor; IQR, interquartile range; LOS, length of stay; OR, odds ratio; CI, confidence interval.

## References

[1] Mattison M.L.P. (2020). Delirium. Ann. Intern. Med..

[2] Salluh J.I., Soares M., Teles J.M., Ceraso D., Raimondi N., Nava V.S., Blasquez P., Ugarte S., Ibanez-Guzman C., Centeno J.V. (2010). Delirium epidemiology in critical care (DECCA): An international study. Crit. Care.

[3] Ely E.W., Inouye S.K., Bernard G.R., Gordon S., Francis J., May L., Truman B., Speroff T., Gautam S., Margolin R. (2001). Delirium in mechanically ventilated patients: Validity and reliability of the confusion assessment method for the intensive care unit (CAM-ICU). JAMA.

[4] Liu S.B., Wu H.Y., Duan M.L., Yang R.L., Ji C.H., Liu J.J., Zhao H. (2024). Delirium in the ICU: How much do we know? A narrative review. Ann. Med..

[5] Juster R.P., Misiak B. (2023). Advancing the allostatic load model: From theory to therapy. Psychoneuroendocrinology.

[6] Fava G.A., McEwen B.S., Guidi J., Gostoli S., Offidani E., Sonino N. (2019). Clinical characterization of allostatic overload. Psychoneuroendocrinology.

[7] McEwen B.S., Stellar E. (1993). Stress and the individual. Mechanisms leading to disease. Arch. Intern. Med..

[8] Guidi J., Lucente M., Sonino N., Fava G.A. (2021). Allostatic Load and Its Impact on Health: A Systematic Review. Psychother. Psychosom..

[9] Chen J.C., Elsaid M.I., Handley D., Plascak J.J., Andersen B.L., Carson W.E., Pawlik T.M., Fareed N., Obeng-Gyasi S. (2024). Association Between Neighborhood Opportunity, Allostatic Load, and All-Cause Mortality in Patients with Breast Cancer. J. Clin. Oncol..

[10] Stabellini N., Cullen J., Bittencourt M.S., Moore J.X., Cao L., Weintraub N.L., Harris R.A., Wang X., Datta B., Coughlin S.S. (2023). Allostatic load and cardiovascular outcomes in males with prostate cancer. JNCI Cancer Spectr..

[11] Sonino N., Fava G.A., Lucente M., Guidi J. (2023). Allostatic Load and Endocrine Disorders. Psychother. Psychosom..

[12] Johnson N.B., Jones E.M., Ovbiagele B., Markovic D., Towfighi A. (2025). Effects of Allostatic Load on Long-Term Survival After Stroke. Stroke.

[13] Guan Y., Shen J., Lu J., Fuemmeler B.F., Shock L.S., Zhao H. (2023). Association between allostatic load and breast cancer risk: A cohort study. Breast Cancer Res..

[14] Duong M.T., Bingham B.A., Aldana P.C., Chung S.T., Sumner A.E. (2017). Variation in the Calculation of Allostatic Load Score: 21 Examples from NHANES. J. Racial Ethn. Health Disparities.

[15] Carbone J.T., Clift J., Alexander N. (2022). Measuring allostatic load: Approaches and limitations to algorithm creation. J. Psychosom. Res..

[16] Maldonado J.R. (2013). Neuropathogenesis of delirium: Review of current etiologic theories and common pathways. Am. J. Geriatr. Psychiatry.

[17] Dilmen O.K., Meco B.C., Evered L.A., Radtke F.M. (2024). Postoperative neurocognitive disorders: A clinical guide. J. Clin. Anesth..

[18] Thomson E.M. (2019). Air Pollution, Stress, and Allostatic Load: Linking Systemic and Central Nervous System Impacts. J. Alzheimers Dis..

[19] Johnson A.E.W., Bulgarelli L., Shen L., Gayles A., Shammout A., Horng S., Pollard T.J., Hao S., Moody B., Gow B. (2023). MIMIC-IV, a freely accessible electronic health record dataset. Sci. Data.

[20] Johnson A., Bulgarelli L., Pollard T., Gow B., Moody B., Horng S., Celi L.A., Mark R. MIMIC-IV (Version 3.0). PhysioNet.

[21] Chen T.J., Chung Y.W., Chang H.R., Chen P.Y., Wu C.R., Hsieh S.H., Chiu H.Y. (2021). Diagnostic accuracy of the CAM-ICU and ICDSC in detecting intensive care unit delirium: A bivariate meta-analysis. Int. J. Nurs. Stud..

[22] International Classification of Diseases, Ninth Revision, Clinical Modification (ICD-9-CM). https://archive.cdc.gov/www_cdc_gov/nchs/icd/icd9cm.htm.

[23] International Classification of Diseases, Tenth Revision, Clinical Modification (ICD-10-CM). https://www.cdc.gov/nchs/icd/icd-10-cm/index.html.

[24] Sapolsky R.M., Romero L.M., Munck A.U. (2000). How do glucocorticoids influence stress responses? Integrating permissive, suppressive, stimulatory, and preparative actions. Endocr. Rev..

[25] McEwen B.S. (2003). Interacting mediators of allostasis and allostatic load: Towards an understanding of resilience in aging. Metabolism.

[26] Juster R.P., McEwen B.S., Lupien S.J. (2010). Allostatic load biomarkers of chronic stress and impact on health and cognition. Neurosci. Biobehav. Rev..

[27] Du E.Y., Jiang K., Carlson M.C., Reed N.S., Deal J.A. (2023). Hearing Impairment and Allostatic Load in Older Adults. JAMA Otolaryngol. Head. Neck Surg..

[28] Wilcox M.E., Girard T.D., Hough C.L. (2021). Delirium and long term cognition in critically ill patients. BMJ.

[29] Wilson J.E., Mart M.F., Cunningham C., Shehabi Y., Girard T.D., MacLullich A.M.J., Slooter A.J.C., Ely E.W. (2020). Delirium. Nat. Rev. Dis. Primers.

[30] Dunne S.S., Coffey J.C., Konje S., Gasior S., Clancy C.C., Gulati G., Meagher D., Dunne C.P. (2021). Biomarkers in delirium: A systematic review. J. Psychosom. Res..

[31] Fong T.G., Inouye S.K. (2022). The inter-relationship between delirium and dementia: The importance of delirium prevention. Nat. Rev. Neurol..

[32] Smith P.J., Attix D.K., Weldon B.C., Greene N.H., Monk T.G. (2009). Executive function and depression as independent risk factors for postoperative delirium. Anesthesiology.

[33] Engel G.L., Romano J. (1959). Delirium, a syndrome of cerebral insufficiency. J. Chronic Dis..

[34] Maclullich A.M., Ferguson K.J., Miller T., de Rooij S.E., Cunningham C. (2008). Unravelling the pathophysiology of delirium: A focus on the role of aberrant stress responses. J. Psychosom. Res..

[35] de Pablos R.M., Villarán R.F., Argüelles S., Herrera A.J., Venero J.L., Ayala A., Cano J., Machado A. (2006). Stress increases vulnerability to inflammation in the rat prefrontal cortex. J. Neurosci..

[36] Palakshappa J.A., Hough C.L. (2021). How We Prevent and Treat Delirium in the ICU. Chest.

[37] Rigney T. (2010). Allostatic load and delirium in the hospitalized older adult. Nurs. Res..

