# Supplementary material for: Association Between Allostatic Load and Delirium in ICU Patients: A Retrospective Analysis of the MIMIC-IV Database
Source: J Clin Med. 2025 Jun 3;14(11):3916. doi: 10.3390/jcm14113916 (PMC12155833; doi:10.3390/jcm14113916)
Supplement: Supplementary file 1 [file jcm-14-03916-s001.zip › jcm-3629991-supplementary.pdf]

Supplementary Materials

Supplementary Table S1. The variance inflation factors for ALS components and the SOFA score

Supplementary Table S2. Result of lasso regression

Supplementary Table S3. The variance inflation factor for variables that have a significant effect on delirium after lasso regression screening

Supplementary Table S4. The association between ALS and risk of delirium in the ICU after excluding patients with sepsis(n=297)

Supplementary Table S5. Outcome of patients with low and high ALS after excluding patients with sepsis (n=297)

Supplementary Table S6. The association between ALS and risk of delirium in the ICU after excluding patients with mechanical ventilation(n=373)

Supplementary Table S7. Outcome of patients with low and high ALS after excluding patients with mechanical ventilation (n=373)

Supplementary Figure S1. Decision Tree for Identifying Optimal ALS Threshold for Delirium.

Table S1. The variance inflation factors for ALS components and the SOFA score

Variable	VIF
ALS (score)	2.240340
SOFA (score)	1.120600
HbA1c(%)	1.181288
HDL(mg/dL)	1.261501
Total cholesterol(mg/dL)	1.247125
ALB(g/dL)	1.382682
CRP (mg/dL)	1.221857
SBP(mmHg)	1.282623
DBP(mmHg)	1.174828
BMI(kg/m^2)	1.161273

Abbreviation: VIF, Variance inflation factor; ALS, allostatic load score; SOFA, Sequential Organ Failure Assessment; HbA1c, hemoglobin A1c; HDL, high-density; CRP, C-reactive protein; ALB, serum albumin; SBP, systolic blood pressure; DBP, diastolic blood pressure; MBP, mean blood pressure; BMI, body mass index

Table S2. Result of lasso regression

Variable	S0
ALS (score)	0.123686959
SOFA (score)	0.017613062
Race (n,%)	-0.367426838
Heart rate (beats/min)	
Respiratory rate (times/min)	0.022295933
Temprature (°C)	0.031912580
Spo2 (%)	0.007435670
Albumin (g/dL)	
WBC (10^9/L)	-0.003803187
Hemoglobin(g/dL)	-0.005810485
Creatinine (mg/dL)	
Blood urea nitrogen (mg/dL)	
AG (mEq/L)	0.032983181
INR	
Sepsis (n,%)	1.067735409
Rheumatic disease (n,%)	-0.683082031
Mechanical ventilation (n,%)	1.085141668

Abbreviations: ALS, allostatic load score; SOFA, the Sequential Organ Failure Assessment; SpO_2, peripheral capillary oxygen saturation; WBC, white blood cell count; AG, anion gap; INR, international normalized ratio.

Table S3. The variance inflation factor for variables that have a significant effect on delirium after lasso regression screening

Variable	VIF
ALS (score)	1.072284
Ethnicity (n,%)	1.037050
SOFA (score)	1.606488
Respiratory rate (times/min)	1.118569
Temprature (°C)	1.137547
SpO_2 (%)	1.129783
WBC (10^9/L)	1.128133
Hemoglobin(g/dL)	1.210424
AG(mEq/L)	1.131900
Sepsis (n,%)	1.405978
Rheumatic disease (n,%)	1.054158
Mechanical ventilation(n,%)	1.375250

Abbreviations: VIF, Variance inflation factor; ALS, allostatic load score; SOFA, the Sequential Organ Failure Assessment; SpO_2, peripheral capillary oxygen saturation; WBC, white blood cell count; AG, anion gap.

Table S4. The association between ALS and risk of delirium in the ICU after excluding patients with sepsis(n=297)

	**Model 1**		**Model 2**	
	OR(95%CI)	*P*-value	OR(95%CI)	*P*-value
ALS[#]	1.22 (0.99, 1.52)	0.064	1.13 (1.00, 1.61)	0.048*
ALS*				
Low risk (≤2)				
High risk (≥3)	2.11 (1.23, 3.62)	0.007*	2.06 (1.12, 3.76)	0.018*

[#] Stands for ALS were a continuous variable. *Stands for the ALS were divided into two groups based on the cutoff values from the decision tree, with the low-risk group used as the reference group.

Model 1: unadjusted model; Model 2: adjusted for race, temperature, SOFA, respiratory rate, SpO_2, WBC, hemoglobin, AG, rheumatic disease, and mechanical ventilation.

Abbreviations: ALS, allostatic load score; OR, odds ratio; CI, confidence interval; SOFA, the Sequential Organ Failure Assessment; SpO_2, peripheral capillary oxygen saturation; WBC, white blood cell count; AG, anion gap. * $p < 0.05$.

Table S5. Outcome of patients with low and high ALS after excluding patients with sepsis (n=297)

Variable	Overall	ALS≤2	ALS≥3	*p*-value
	297	215	82	
Deliruim (n, %)	85 (28.6)	52 (24.2)	33 (40.2)	0.009*
Reintubation within 48h (n, %)	2 (0.67)	1 (0.47)	1 (1.21)	1
ICU LOS (day)	2.92 (1.78, 4.77)	2.86 (1.75, 4.89)	3.02 (1.94, 4.62)	0.945
Hospital LOS (day)	9.72 (4.90, 18.0)	8.17 (4.11, 17.5)	11.5 (6.66, 19.2)	0.003*
In-hospital death (n, %)	19 (6.40)	14 (6.51)	5 (6.10)	1
Mechanical ventilation (n, %)	65 (21.89)	43 (20)	22 (26.8)	0.265

Abbreviations: ALS, allostatic load score; ICU, intensive care unit; LOS, length of stay. * $p < 0.05$.

Table S6. The association between ALS and risk of delirium in the ICU after excluding patients with mechanical ventilation(n=373)

	**Model 1**		**Model 2**	
	OR(95%CI)	*P*-value	OR(95%CI)	*P*-value
ALS[#]	1.30 (1.09, 1.56)	0.004*	1.24 (1.02, 1.51)	0.034*
ALS*				
Low risk (≤2)				
High risk (≥3)	2.14 (1.36, 3.36)	<0.001*	1.76 (1.07, 2.90)	0.025*

[#] Stands for ALS was a continuous variable. *Stands for the ALS were divided into two groups based on the cutoff values from the decision tree, with the low-risk group used as the reference group.

Model 1: unadjusted model; Model 2: adjusted for race, temperature, SOFA, respiratory rate, SpO_2, WBC, hemoglobin, AG, rheumatic disease, and sepsis.

Abbreviations: ALS, allostatic load score; OR, odds ratio; CI, confidence interval; SOFA, the Sequential Organ Failure Assessment; SpO_2, peripheral capillary oxygen saturation; WBC, white blood cell count; AG, anion gap. * $p < 0.05$.

Table S7. Outcome of patients with low and high ALS after excluding patients with mechanical ventilation (n=373)

Variable	Overall	ALS≤2	ALS≥3	*p*-value
	373	255	118	
Delirium (n, %)	129 (34.58)	74 (29)	55 (46.6)	0.001*
ICU LOS (day)	3.16 (1.95, 5.75)	3.11 (1.89, 5.75)	3.26 (2.16, 5.59)	0.747
Hospital LOS (day)	11.1 (5.97, 20.1)	10.7 (4.77, 18.1)	13.9 (7.91, 27.4)	< 0.001*
In-hospital death (n, %)	25 (6.70)	16 (6.27)	9 (7.63)	0.792

Abbreviations: ALS, allostatic load score; ICU, intensive care unit; LOS, length of stay. * $p < 0.05$.

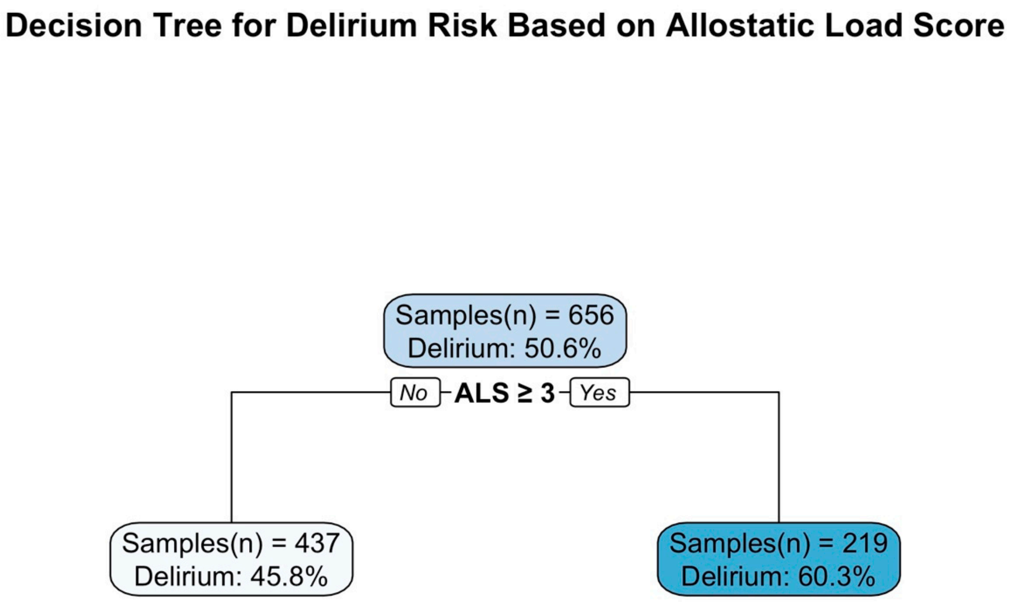

Figure S1. Decision Tree for Identifying Optimal ALS Threshold for Delirium. A decision tree illustrating how ALS stratifies delirium risk. The primary split occurs at ALS = 3, identifying two groups with distinct risk levels. Color coding differentiates risk levels, with blue indicating higher delirium risk. Abbreviation: ALS, allostatic load score
